# Relationship of biofilm formation and different virulence genes in uropathogenic Escherichia coli isolates from Northwest Iran

**DOI:** 10.3205/dgkh000254

**Published:** 2015-07-13

**Authors:** Sargol Fattahi, Hossein Samadi Kafil, Mohammad Reza Nahai, Mohammad Asgharzadeh, Roghaya Nori, Mohammad Aghazadeh

**Affiliations:** 1 Infectious Disease and Tropical Medicine Research Center, Tabriz University of Medical Sciences, Tabriz, Iran; 2 Drug Applied Research Center, Tabriz University of Medical Sciences, Tabriz, Iran; 3 Biotechnology Research Center, Tabriz University of Medical Sciences, Tabriz, Iran; 4 Immunology Research Center, Tabriz University of Medical Sciences, Tabriz, Iran

**Keywords:** Escherichia coli, fimA, papC, hyl, urinary tract infection, biofilm

## Abstract

**Background and objectives:** The *Escherichia coli* (*E. coli*) bacterium is one of the main causative agents of urinary tract infections (UTI) worldwide. The ability of this bacterium to form biofilms on medical devices such as catheters plays an important role in the development of UTI. The aim of the present study was to investigate the possible relationship between virulence factors and biofilm formation of *E. coli* isolates responsible for urinary tract infection.

**Materials and methods:** A total of 100 *E. coli* isolates isolated from patients with UTI were collected and characterized by routine bacteriological methods. In vitro biofilm formation by these isolates was determined using the 96-well microtiter-plate test, and the presence of *fimA*, *papC*, and *hly* virulence genes was examined by PCR assay. Data analysis was performed using SPSS 16.0 software.

**Results:** From 100 *E. coli* isolates isolated from UTIs, 92% were shown to be biofilm positive. The genes *papC*, *fimA*, and *hly* were detected in 43%, 94% and 26% of isolates, respectively. Biofilm formation in isolates that expressed *papC*, *fimA*, and *hly* genes was 100%, 93%, and 100%, respectively. A significant relationship was found between presence of the *papC* gene and biofilm formation in *E. coli* isolates isolated from UTI (*P*<0.01), but there was no statistically significant correlation between presence of *fimA* and *hly* genes with biofilm formation (*P*<0.072, *P*<0.104).

**Conclusion:** Results showed that *fimA* and *hly* genes do not seem to be necessary or sufficient for the production of biofilm in *E. coli*, but the presence of *papC* correlates with increased biofilm formation of urinary tract isolates. Overall, the presence of *fimA*, *papC*, and *hly* virulence genes coincides with in vitro biofilm formation in uropathogenic *E. coli* isolates.

## Zusammenfassung

**Hintergrund und Zielsetzung:** *Escherichia* (E.) *coli* ist weltweit einer der wichtigsten Erreger von Harnweginfektionen (HWI). Seine Fähigkeit zur Biofilmbildung auf Devices wie Harnwegkatheter spielt eine wichtige Rolle bei der Entstehung von HWI. Zielsetzung der Studie war die Untersuchung des möglichen Zusammenhangs zwischen Virulenzfaktoren und Biofilmbildung bei *E. coli-*Isolaten von HWI.

**Material und Methode:** 100 *E. coli-*Isolate von Patienten mit HWI wurden bakteriologisch charakterisiert. Die Biofilmbildung wurde in vitro in 96-Well-Microtiterplatten und das Vorkommen von *fimA*-, *papC*- und *hly*-Virulenzgenen mittels PCR untersucht. Die Datenanalyse wurde mittels SPSS 16.0 Software durchgeführt.

**Ergebnisse:** 92 der 100 *E. coli-*Isolate zeigten die Fähigkeit zur Biofilmbildung. Die Virulenzgene *papC*, *fimA* und *hly* wurden in 43%, 94% bzw. 26% der Isolate nachgewiesen. Die Biofilmbildung in Isolaten, die *papC*-, *fimA-* und *hly-*Gene exprimierten, betrug 100%, 93% bzw. 100%. Es konnte ein signifikanter (p<0.01) Zusammenhang zwischen dem Vorkommen des *papC-*Gens und der Biofilmbildung nachgewiesen werden, während keine signifikante Korrelation zum Vorkommen des *fimA-* und *hly-*Gens nachweisbar war (p<0.072 bzw. p<0.104).

**Schlussfolgerungen:** Offensichtlich sind das *fimA-* und *hly-*Gen nicht zur Biofilmbildung von *E. coli* erforderlich, während das Vorkommen des *papC-*Gens mit erhöhter Biolfilmbildung korreliert. Insgesamt kamen bei *E. coli-*Isolaten mit der Fähigkeit zur Biofilmbildung gleichzeitig die Virulenzgene *fimA*, *papC* und *hly* vor.

## Introduction

Urinary tract infections (UTIs) are a major public health concern in developed countries and also represent one of the most common nosocomial infections. Worldwide, about 150 million people are diagnosed with UTI each year. The primary etiological agent of UTI is *E. coli* (80%), followed by the much less common *Staphylococcus saprophyticus* and occasionally *Klebsiella*, *Proteus* or other Gram-negative bacteria. Up to 40% of women will develop UTI at least once during their lives, and a significant number of these women will have recurrent UTIs [1], [2], [3], [4], [5]. The severity of UTI depends on the virulence of the bacterium and host sensitivity [4].

Uropathogenic* Escherichia coli* (UPEC) has several virulence factors that allow it to colonize host mucosal surfaces, injure and invade host tissues, overcome hosts defense mechanisms and incite a host inflammatory response [5]. UPEC can colonize the bladder through the urinary tract and cause cystitis; this organism is also able to move through the ureters to the kidneys and cause pyelonephritis [6]. *E. coli* isolates isolated from UTI often express specific virulence properties that are not prevalent among isolates from normal fecal flora. The *E. coli* virulence factors (VFs) that cause urinary tract infection include adhesins, α-hemolysin (Hly), cytotoxic necrotizing factor, fimbriae, aerobactin-mediated iron uptake, K1 capsular polysaccharide, and biofilm formation. These factors ultimately lead to tissue damage [7], [8]. The ability of bacteria to attach to uroepithelial cells through specific fimbriae and adhesions is critical for the initiation of infection [9]. Biofilms are sticky accumulations of small colonies of bacteria surrounded by an extracellular polysaccharide matrix in which cell aggregations adhere to various surfaces, including medical devices and injured tissues [10]. 

A hallmark of UPEC is the formation of biofilm, which facilitates the persistence of these pathogenic isolates in the urinary tract and interferes with bacterial eradication. Biofilm infections are difficult to eradicate with antimicrobial treatment, and in vitro susceptibility tests show considerable resistance of biofilm cells to killing [11], [12], [13]. There is no basis for proposing the existence of biofilm resistance mechanisms, but it is clear that a large number of biofilm-constitutive microorganisms are highly resistant to antimicrobial agents [14], [15]. Biofilm formation in *E. coli* requires a set of gene expressions facilitating its initiation, attachment, and subsequent maturation. A variety of virulence factors are involved in biofilm formation in *E. coli*, including hemolysin, fimbriae, lipopolysaccharides (LPS), secreted proteins, capsules, and iron-acquisition systems, which allow attachment and bacterial colonization in the mucosal epithelial cells lining the urinary tract, invading and further forming intracellular biofilm-like pods in uroepithelial cells.

Three main virulence determinants of UPEC isolates are involved in biofilm formation: type 1 fimbriae (*fim*), coded by the *fim* gene cluster: the P-fimbriae (*pap*), coded by the *pap* (pyelonephritis-associated pili) gene; and α-hemolysin (*hly*), a member of the RTX toxin family on the basis of a common nonapeptide repeat in the C-terminal part of the protein, that were investigated in the present study [16], [17]. 

It is known that α-haemolysin acts on a wide range of host cells, such as the erythrocytes of mammals, birds and fish, embryonic fibroblasts, mouse fibroblasts, monocytes, polymorphonuclear leukocytes, and macrophages [18]. Biofilms are probably the usual phenotypic state of microorganisms in natural environments and they are regularly involved in infections associated with biomaterials, such as urinary catheters. Production of a biofilm may be considered as another pathogenic determinant which allows the bacteria to persist for long periods in the urogenital tract and interferes with bacterial eradication due to the expression of virulence and resistance genes. 

The aim of the present study was to determine the relationship between biofilm formation and possibly related virulence factors of *E. coli* isolates isolated from UTI patients. Biofilm formation in UPEC with the simultaneous presence of associated virulence genes can be targeted in the treatment of the patients affected.

## Materials and methods

This cross-sectional study was conducted from July to December 2014 in Northwest Iran. All isolates were characterized by using the bacterial identification system API 20 E (Bio Merieux, Marcy d’Etoile, France). A total of 100 *E. coli* isolates were isolated and collected from urine specimens of patients with UTI who were hospitalized in the Imam Reza hospital (Tabriz), in the Northwest of Iran. All the characterized bacteria were preserved in TSB (Tryptic soy broth) containing 15% glycerol at –70°C [19].

### Biofilm assay

For each isolate, a few colonies were suspended in a test tube containing 5 ml of Luria Brtany (LB) and this incubated at 37°C for 18 to 24h. After this period, 1 ml of bacterial suspension was inoculated into a different test tube containing 10 ml of sterile LB medium. Three wells of a sterile 96-well flat-bottomed plastic tissue culture plate with a lid (Greiner bio-one GmbH, Germany) were filled with 200 ml of each bacterial suspension. Negative controls (blank) were LB broth alone, which were dispensed into eight wells per tray. After stationary aerobic incubation at 37°C for 24h, the content of the wells was carefully drawn off and each well was washed three times with 250 ml of sterile physiological saline. The plates were shaken to remove all non-adherent bacteria [19].

The remaining attached bacteria (biofilm) in the wells were fixed with 200 ml of methanol 99% (Merck, Germany) for 15 min, which was then discarded. The wells were flicked and let air dry in an inverted position (room temperature, about 30 min).

Biofilms were stained with 0.2 ml of crystal violet 2% (used for Gram staining) for 5 min at room temperature. Excess stain remaining in the wells was rinsed out by placing the plate under running tap water. Afterwards, the plates were inverted on a towel and let air dry.

The dye bound to the adherent cells in the each well was resolubilized with 160 ml of glacial acetic acid 33% (Merck, Germany) to quantifiy biofilm production. The lidded plates were left at room temperature for 30 min [19]. 

Afterward, the optical density (OD) of resolubilized crystal violet in each well was measured at 570 nm (OD570) using an Elisa reader. The reading was performed twice: (i) before addition of glacial acetic acid, as a standard microtiter-plate test and (ii) after addition of glacial acetic acid. For a comparative analysis of test results, we introduce a classification of adherence capabilities of tested isolates into four categories. 

All isolates were classified into the following categories: non-adherent (-), weakly (+), moderately (++) and strongly (+++) adherent, based on the ODs of bacterial biofilms.

The cutoff OD value (ODc) was defined as three standard deviations above the mean OD of the negative control. Isolates were classified as follows:

OD ≤ ODc = not a biofilm producer (-),

ODc < OD ≤ 2×ODc = weak biofilm producer (+),

2×ODc < OD ≤ 4×ODc = moderate biofilm producer (++),

4×ODc < OD = strong biofilm producer (+++) [20].

All tests were carried out 3 times and the results were averaged.

### DNA extraction and PCR method

Total genomic DNA was extracted from all preserved isolates (100 *E.coli* isolates) using a DNA extraction kit (Bioneer, South Korea) according to the manufacturer’s instructions.

Amplification and detection of the considered genes encoding *VFs* (*hly, fimA *and* papC *genes) was done by the PCR method using specific primers (Table 1 (Tab. 1)).

The presence of the *papC* and *hly* genes was established by use of the conditions described by Yamamoto et al. [21]. The PCR conditions used to amplify a fragment of the *fimA* gene were those explained by Vila et al. [22], although 35 cycles instead of 30 cycles were used. Ultimately, the conditions are described by Vargas et al. [23] and Vila et al. [22]. All samples with negative results were retested at least twice to eliminate the possibility of false-negative results. The several isolates with positive PCR results randomly selected of the entire samples and recovered and their sequences were determined and analyzed using an automatic DNA sequencer (Termocycle, Bioneer, Korea), in order to perform a quality control for the PCR products obtained.

### Statistical analysis

Statistical analysis was performed using SPSS software for Windows, version 16 (SPSS 16.0). Chi-square or Fisher’s exact test was used to evaluate the relationship between the variables. The level of significance was set at p<0.05.

## Results

**Figure 1:**
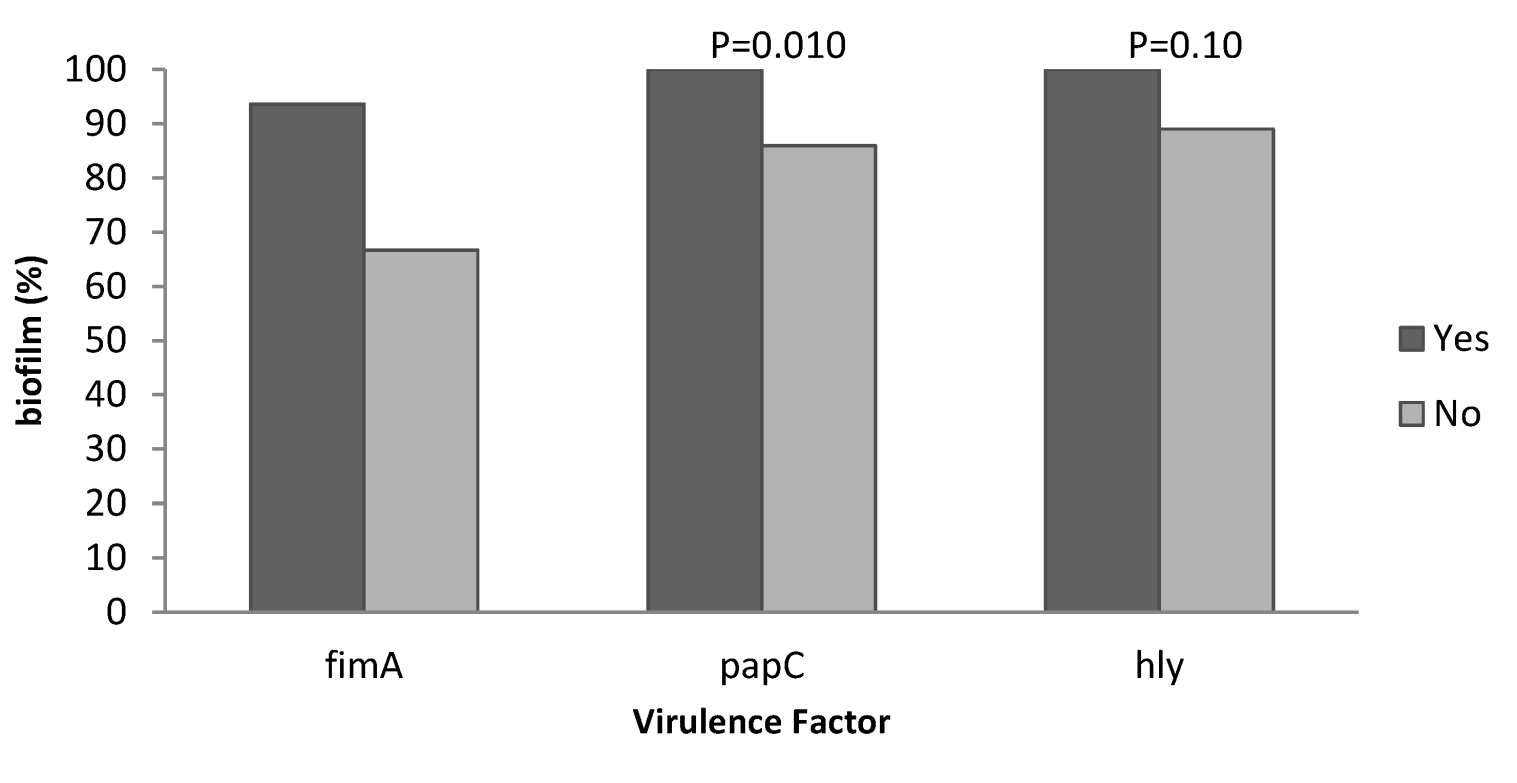
Relationship between expression of *fimA*, *papC* and *hly* and biofilm formation using Fisher's exact test (N=100)

Polymerase chain reaction showed that the prevalence of virulence genes in 100 UPEC strains was 43%, 27%, and 94% for *papC*, *hly*, and *fimA* genes, respectively. Biofilm production assay showed that 92% of the total isolates were biofilm producers. A summary of results of microtiter-plate tests is presented in Table 2 (Tab. 2). Of 43 *papC* positive isolates, all of them were biofilm producers. This shows that there was a strong relationship between the presence of *papC* virulence genes and biofilm formation in UPEC isolates (*P*<0.01), but these findings do not apply to the two other genes (*fimA* and *hly*). There was no statistically significant correlation between the presence of *fimA* and *hly* genes and biofilm formation (*P*<0.072, *P*<0.104). The relationship between biofilm production and expression of *papC*, *fimA* and *hly* genes by use of Fisher’s exact test is described in Table 3 (Tab. 3). The statistical analysis of biofilm formation and the presence of virulence genes is shown in Figure 1 (Fig. 1).

## Discussion

*E. coli* is the most frequent cause of urinary tract infections. There are several virulence factors in UPEC isolates that increase their ability to colonize and persist in the urogenital tract [5], [24]. The severity of UTI is dependent on bacterial virulence factors and host susceptibility. *E. coli* VFs causing urinary tract infection include a-hemolysin (Hly), cytotoxic necrotizing factor, fimbriae, iron-acquisition systems (aerobactin), biofilm formation [24], [25]. The ability of bacteria form biofilms on medical devices, e.g. catheters, is believed to play a major role in the development of nosocomial infections, including catheter-associated urinary tract infections [26], [27], [28]. 

Biofilm formation has been described as an important virulence factor in various pathogenic bacteria causing human UTI. In the study conducted by Jabalameli et al. [29], it was shown that biofilm formation in *Pseudomonas aeruginosa* isolates was seen in more than 96% of isolates, where 47% of isolates were strong, 26% were moderate and 22.9% were weak biofilm producers. In the present study, biofilm formation was seen in 100% of isolates, of which 48.6% were strong, 11.4% were moderate and 40% were weak biofilm producers [29]. A high prevalence of *fimA* and *papC* genes in the present study is consistent with results of previous studies by other researchers [30], [31]. The rate of biofilm formation in isolates that expressed the *fimA* gene was 93/6% which was higher than in those that did not express this gene with 66/7% (p=0.072); and the rate of cases in which the *hly* gene was expressed in biofilms was 100% which was tendentially higher (p=0.104) than those without *hly* gene expression, so there is not significantly relationship between presence of these genes and biofilm production. In a study by Soto et al. [5], biofilm-producing *E. coli* isolates isolated from the urinary tract and causing inflammation of prostate were often the highest hemolysin-containing isolates, and biofilm production was significantly correlated with the expression of type I fimbriae and hemolysin.

In the study conducted by Tarchouna et al. [32], the distribution of virulence genes in *E. coli* isolated from patients with UTI, screened by PCR for the prevalence of virulence genes encoding pili, was associated with pyelonephritis (*pap*) and hemolysin (*hly*). The prevalence of genes coding *pap* and *hly* were 41% and 19%, respectively.

In a study by Naves et al. [33], five virulence-associated genes were involved in strong biofilm production, including: *papC* and *papG* alleles, *sfa/focDE*, *focG*, *hlyA* and *cnf1*, with two of these *(papC* and *hlyA)* being more common (*p*<0.05) than the others. Biofilm formation where the *papC* gene was expressed (100%) was significantly higher than in cases where the *papC* gene was not expressed (86%) (p=0.010). In a study conducted by Farshad et al. [34], the expression of α-hemolysin (*hlyA*) was 15.6%, but in the present study, the expression of this gene was 27%. The results of these two studies nearly were similar, and the differences in result could be due to differences in the study population. Our study showed that the prevalence of virulence genes *fimA*, *papC*, and *hly*, as well as isolates of UPEC in hospitalized patients in the study area is high, as is the formation of biofilms.

The results of the present and previous study demonstrated a significant association between virulence gene expression involved in bacterial attachment and biofilm formation, indicating that biofilm-forming bacteria are more pathogenic than the planktonic form in urinary tract infections, and that biofilm formation cause increasing the rate of bacterial virulence as well as the severity of disease, making biofilm-based UTI very difficult to cure.

Different results in various studies may be due to regional differences in hygiene status and increasing resistance to antibiotics. The limitations of the present study are that 1. The number of evaluated genes involved in virulence and biofilm formation was lower than in other studies and 2. the targeted community in this study was selected from one specific geographical area. These may explain the difference in results compared to other studies.

## Conclusion

The results of the current study showed that *fimA* and *hly* genes do not seem to be necessary or sufficient for the production of biofilm in *E. coli* isolates, but the expression of the *papC* gene is correlated to increased biofilm formation in UPEC. Overall, the presence of *fimA*, *papC*, and *hly* virulence genes is concomitant with in vitro biofilm formation in isolates, so it can be used as a target in the treatment of affected patients. Further investigation of virulence genes associated with biofilm formation is necessary for effective treatment of patients with antibiotic-resistant UTI.

## Notes

### Competing interests

The authors declare that they have no competing interests.

### Acknowledgments

We thank the staff of Imam Reza, Madani, Sina and 29 Bahman Hospitals for their collaboration in sample collecting. This study was supported by a grant from the Immunology Research Center, Tabriz University of Medical Sciences and was conducted as the Msc thesis of Sargol Fattahi. 

## Figures and Tables

**Table 1:**
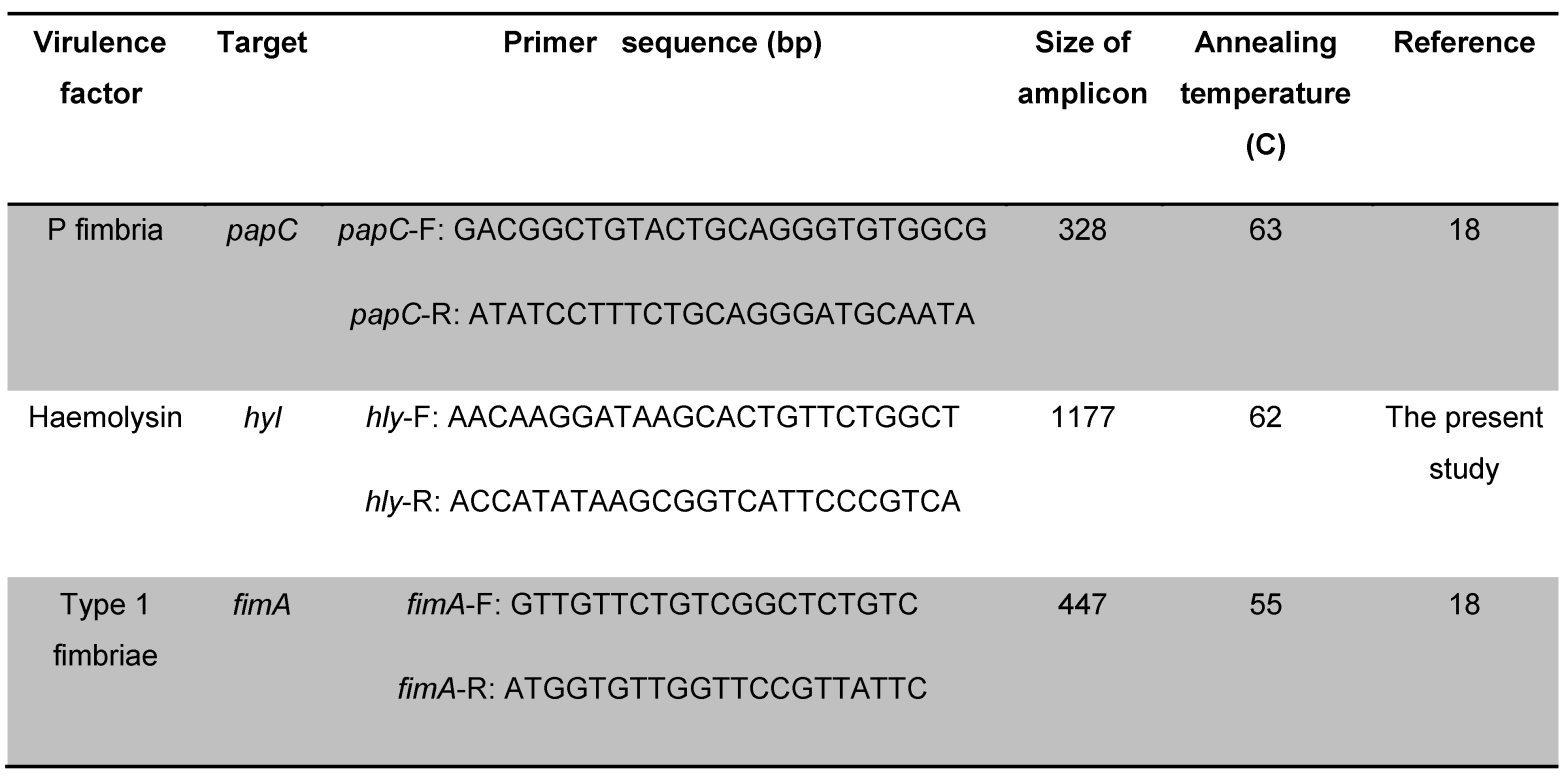
Primers used for amplification of the virulence genes examined here using PCR assays

**Table 2:**
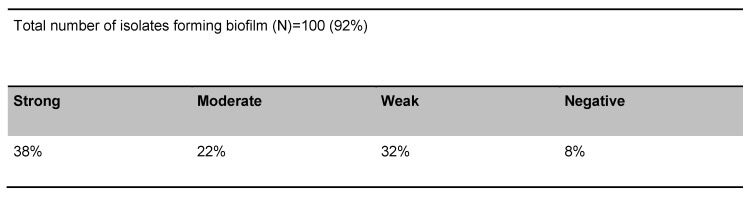
Biofilm formation in *E.coli* isolates by microtiter-plate tests

**Table 3:**
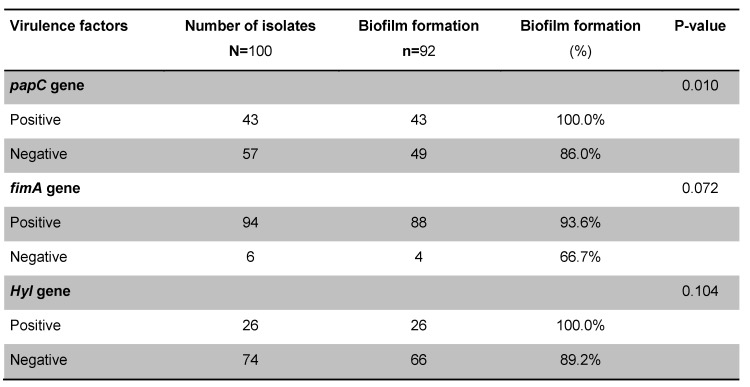
Relationship between biofilm formation and expression of virulence factors in 100 *E. coli* isolates using Fisher's exact test
